# Mediators of the effects of rice intake on health in individuals consuming a traditional Japanese diet centered on rice

**DOI:** 10.1371/journal.pone.0185816

**Published:** 2017-10-02

**Authors:** Minori Koga, Atsuhito Toyomaki, Akane Miyazaki, Yukiei Nakai, Atsuko Yamaguchi, Chizuru Kubo, Junko Suzuki, Iwao Ohkubo, Mari Shimizu, Manabu Musashi, Yoshinobu Kiso, Ichiro Kusumi

**Affiliations:** 1 Department of Psychiatry, Hokkaido University Graduate School of Medicine, Sapporo, Japan; 2 Department of Nutrition, Tenshi College, Sapporo, Japan; 3 Institute for the Promotion of Business-Regional Collaboration, Hokkaido University, Sapporo, Japan; The University of Tokyo, JAPAN

## Abstract

Although the Japanese diet is believed to be balanced and healthy, its benefits have been poorly investigated, especially in terms of effects on mental health. We investigated dietary patterns and physical and mental health in the Japanese population using an epidemiological survey to determine the health benefits of the traditional Japanese diet. Questionnaires to assess dietary habits, quality of life, sleep quality, impulsivity, and depression severity were distributed to 550 randomly selected middle-aged and elderly individuals. Participants with any physical or mental disease were excluded. Two-hundred and seventy-eight participants were selected for the final statistical analysis. We determined rice to be one of the most traditional foods in Japanese cuisine. Scores for each questionnaire were computed, and the correlations between rice intake and health indices were assessed. When analyzing the direct correlations between rice intake and health indices, we found only two correlations, namely those with quality of life (vitality) and sleep quality. Path analysis using structural equation modeling was performed to investigate the association between rice intake and health, with indirect effects included in the model. Additional associations between rice intake and health were explained using this model when compared to those using direct correlation analysis. Path analysis was used to identify mediators of the rice-health association. These mediators were miso (soybean paste) soup, green tea, and *natto* (fermented soybean) intake. Interestingly, these mediators have been major components of the Japanese diet since 1975, which has been considered one of the healthiest diets since the 1960s. Our results indicate that the combination of rice with other healthy foods, which is representative of the traditional Japanese diet, may contribute to improvements in physical and mental health.

## Introduction

Traditional Japanese cuisine, also known as Washoku, was registered by the United Nations Educational, Scientific, and Cultural Organization in 2013. Currently, the Japanese diet is popular as a healthy diet in many countries. However, scientific evidence regarding the effects of the Japanese diet on health is poorly understood. Since World War II, the lifestyle of Japanese people has become increasingly westernized, and the dietary pattern is no exception; consequently, the composition of nutritional intake has changed [1]. In parallel, patterns of causes of death have changed, such as an increase in the incidence of cardiac diseases and cancer [1, 2]. Thus, changes in dietary habits may be associated with changes in health outcomes in Japan. A number of reports in basic and clinical studies indicate that dietary pattern impacts brain health [3].

To demonstrate the health benefits of the Japanese diet, we focused on the physical and mental health benefits of rice, which is a traditional and primary staple food in Japan [4]. A recent study showed rice intake was associated with sleep quality, due to specific nutritional factors in rice [5]. Rice is a major staple food in Japan, and the traditional Japanese diet has consisted of rice-centered dishes developed through a long history in Japan [6]. In the present study, we assessed dietary patterns and physical and mental health using questionnaires to examine the associations of rice-centered cuisine with health. We hypothesized that there would be associations between rice intake and improvements in health and that this association would potentially be modified by other healthy foods.

## Materials and methods

### Study participants

The subjective questionnaire survey was administered to 550 randomly selected men and women over 40 years of age. The response rate for the survey was 63.6%. Participants with unrealistic dietary energy intakes (i.e., less than 500 kcal or more than 4,000 kcal) and those with any physical or mental diseases were excluded. Two-hundred and seventy-eight participants (169 men and 109 women) were included in the final analysis. The demographic characteristics of the study participants are shown in Table 1. Written informed consent was obtained from all participants. The study was approved by the Institutional Ethical Board for Epidemiological Studies at the Hokkaido University Graduate School of Medicine (approval number, 14–059).

**Table 1:** Demographic characteristics of the study population. Smoking includes current and past experience.

Characteristics	Participants
N	278
Sex (male, %)	60.8
Age (mean ± standard deviation)	48.8 ± 7.5
Body mass index (mean %)	22.7 ± 2.9
Smoking (yes, %)	21.9
Alcohol use (yes, %)	80.2
Regular physical activity (yes, %)	79.5

### Questionnaires

To survey the frequency of staple food intake in the participants, they were asked the following question: “What is the staple food in your meals: rice, rice-based foods, bread, noodles, cereal, or other?” The participants provided their responses for the three main meals of the day (breakfast, lunch, and dinner). The number of times rice and bread were listed were counted and used as the scores for rice and bread frequency for the three daily meals.

Dietary patterns were characterized using the original questionnaire for the dietary pattern and the Brief-type Self-administered Diet History Questionnaire (BDHQ) [7]. The BDHQ consists of 75 questions used to assess food consumption and cooking patterns over the past month. Energy and food intakes were calculated using an ad hoc computer algorithm [7].

Depression was assessed using the Japanese version of the Patient Health Questionnaire (PHQ-9) [8]. This questionnaire was originally established to screen for and diagnose depression with the threshold of a PHQ-9 score of 10. In addition to the use of criteria-based diagnoses for depressive disorders, the PHQ-9 has been validated as a reliable tool for measuring depression severity [9]. PHQ-9 scores correlate with depression on the following scale: none (0 to <5), mild (5 to <10), moderate (10 to <15), moderately severe (15 to <20), and severe (>20).

Impulsiveness was assessed using the Japanese version of the Barratt Impulsiveness Scale, 11th version (BIS-11) [10]. The BIS-11 consists of 30 questions used to assess impulsiveness. Each item is measured on a 4-point scale (rarely or never [1] to almost or always [4]), where higher scores indicate higher impulsiveness. The questions on the BIS-11 can be placed into one of four categories: lack of self-control, lack of deliberate thinking, lack of thinking/planning, and impulsiveness.

Quality of life as a measure of health was assessed using the Japanese version of Short Form 8 (SF-8^™^) [11], which is a 24-hour recall questionnaire with eight questions used to assess intentions to carry out protective behaviors. Each item is measured on a 5- or 6-point scale (1, the best condition; 5 or 6, the worst condition). The questionnaire can be summarized into eight health dimensions, general health perception (GH), role functioning–physical (RP), bodily pain (BP), physical functioning (PF), vitality (VT), social functioning (SF), mental health (MH), and role functioning–emotional (RE); and two summary measures, a physical component summary (PCS), and a mental component summary (MCS).

Sleep quality was measured using the Japanese version of the Pittsburgh Sleep Quality Index (J-PSQI) [12]. The questionnaire comprises seven domains: subjective sleep quality, latency, duration, sleep efficiency, nighttime disturbances, sleep medication use, and daytime dysfunction. The J-PSQI score is the sum of all sub-scores from the seven domains. Higher scores reflect more frequent sleep disturbance, which indicates lower sleep quality.

### Statistical analyses

Food intake frequency and amount were normalized for sex and age, and Pearson's correlation was used to determine whether there were substantial relationships between dietary patterns and health survey exam scores. We used JMP Pro 12 (SAS Institute Inc., North Carolina) or IBM SPSS version 22 (IBM Corp., Armonk, NY) to perform the statistical analyses. Structural equation modeling (SEM) was performed to determine causal relationships between food consumption, food intake, and physical and mental health, as determined by scores on the above-described tests. Root Mean Square Error of Approximation (RMSEA), comparative fit index (CFI), and Tucker-Lewis Index (TLI) were used to analyze the fit of the model. SEM, RMSEA, CFI, and TLI were calculated using the Mplus Program, Version 7.3 (Muthén and Muthén, 2014). Values less than 0.1 for RMSEA and values above 0.90 for CFI and TLI were indicative of a model with good fit.

## Results

### Associations of characteristics of the study participants with health indices

Scores on the SF-8 (GH, RP, BP, and PCS) and BIS-11 (deliberate thinking, self-control, thinking/planning, and total score) had significant correlations with sex. Scores on the SF-8 (BP and MH) had significant correlations with age. Body mass index (BMI) and daily alcohol consumption did not have significant correlations with any of the health indices investigated in the present study. Smoking experience had a significant correlation with the deliberate thinking score on the BIS-11 (Table 2).

**Table 2:** Scores of health indices according to the profiles of the participants. Sex differences were assessed by t-test, and differences in smoking were assessed by Tukey-Kramer honest significant different test. Correlations between age, BMI, and alcohol consumption were assessed using Pearson's tests. BMI, body mass index (height (m^2^) / body weight (kg)). Alcohol consumption (g/day per 1,000 kcal of daily consumption) was calculated as a part of the BDHQ.

	SF-8 GH	SF-8 PF	SF-8 RP	SF-8 BP	SF-8 VT	SF-8 SF
Sex						
Male, mean (SD)	50.11 (6.10)	51.05 (4.35)	51.80 (3.97)	51.26 (7.76)	51.28 (5.82)	50.75 (6.47)
Female, mean (SD)	49.76 (5.99)	49.91 (5.94)	50.10 (5.22)	49.22 (8.81)	50.80 (5.74)	49.28 (7.12)
P value (t-test)	0.63	**0.07**	**0.002**	**0.04**	0.5	0.08
Age						
P value (Pearson’s test)	0.75	0.52	0.34	**0.041**	0.24	0.86
BMI						
P value (Pearson’s test)	0.5	0.09	0.4	0.14	0.76	0.75
Alcohol consumption						
P value (Pearson’s test)	0.58	0.8	0.65	0.53	0.31	0.98
Smoking						
Never, mean (SD)	49.79 (6.19)	50.97 (4.32)	51.23 (4.46)	50.91 (8.23)	50.71 (6.03)	49.12 (0.60)
Past, mean (SD)	50.44 (6.18)	49.99 (6.08)	50.81 (4.72)	50.12 (8.36)	51.53 (5.53)	51.27 (0.71)
Current, mean (SD)	49.69 (5.62)	50.77 (4.77)	51.39 (4.60)	49.40 (8.08)	51.24 (5.67)	50.68 (0.83)
P value (Tukey-Kramer test)	0.68	0.36	0.7	0.49	0.57	0.06
	SF-8 MH	SF-8 RE	SF-8 PCS	SF-8 MCS	BIS-11 Impulsive	BIS-11 Deliberate
Sex						
Male, mean (SD)	49.45 (6.18)	50.23 (5.14)	50.61 (5.52)	48.81 (6.08)	13.84 (3.32)	8.62 (2.04)
Female, mean (SD)	48.71 (6.26)	50.08 (5.23)	48.90 (6.69)	48.70 (6.35)	14.56 (3.09)	9.65 (2.32)
P value (t-test)	0.34	0.82	**0.02**	0.88	0.07	**0.0001**
Age						
P value (Pearson’s test)	**0.0037**	0.54	0.6	0.17	0.45	0.69
BMI						
P value (Pearson’s test)	0.39	0.49	0.051	0.11	0.23	0.8
Alcohol consumption						
P value (Pearson’s test)	0.62	0.93	0.69	0.89	0.28	0.91
Smoking						
Never, mean (SD)	49.11 (6.24)	50.03 (5.28)	50.26 (6.09)	48.17 (6.71)	13.94 (3.08)	9.22 (2.38)
Past, mean (SD)	49.31 (6.11)	50.37 (5.13)	49.69 (6.55)	49.45 (5.60)	14.45 (3.45)	8.70 (2.11)
Current, mean (SD)	49.07 (6.40)	50.15 (5.03)	49.71 (5.23)	48.96 (5.85)	14.05 (3.30)	9.13 (1.97)
P value (Tukey-Kramer test)	0.97	0.9	0.75	0.32	0.51	0.22
	BIS-11 Selfcont	BIS-11 Thinking	BIS-11 Sum	PHQ-9	PSQI-J	
Sex						
Male, mean (SD)	9.43 (2.30)	8.78 (1.80)	40.64 (6.72)	2.23 (2.82)	4.14 (2.35)	
Female, mean (SD)	10.25 (2.25)	10.12 (1.81)	44.79 (6.35)	3.23 (3.21)	4.06 (2.57)	
P value (t-test)	**0.0037**	**<0.0001**	**<0.0001**	**0.0068**	0.8	
Age						
P value (Pearson’s test)	0.78	0.59	0.43	0.21	0.62	
BMI						
P value (Pearson’s test)	0.57	0.06	0.97	0.09	0.55	
Alcohol consumption						
P value (Pearson’s test)	0.08	0.67	0.32	0.2	0.43	
Smoking						
Never, mean (SD)	9.39 (2.45)	9.40 (2.01)	41.96 (7.02)	2.69 (2.96)	4.17 (2.59)	
Past, mean (SD)	9.90 (2.33)	9.08 (1.76)	42.25 (7.11)	2.57 (2.99)	3.80 (2.14)	
Current, mean (SD)	10.24 (1.92)	9.44 (1.94)	42.83 (6.31)	2.56 (3.21)	4.44 (2.48)	
P value (Tukey-Kramer test)	**0.04**	0.41	0.72	0.94	0.26	

SD, standard deviation; SF-8 GH, general health; SF-8 PF, physical functioning; SF-8 RP, role—physical; SF-8 BP, bodily pain; SF-8 VT, vitality; SF-8 SF, social functioning; SF-8 MH, mental health; SF-8 RE, role—emotional; SF-8 PCS, physical component summary; SF-8 MCS, mental component summary: BIS-11, Barratt Impulsiveness Scale-11; BIS-11 Impulsive, subscale of impulsivity; BIS-11 Deliberate, subscale of lack of planning; BIS-11 Selfcont, subscale of lack of self-control; BIS-11 Thinking, subscale of non-planning impulsivity; BIS-11 sum, sum of subscales of BIS-11; PHQ9, Patient Health Questionnaire-9; PSQI-J, Pittsburgh Sleep Quality Index, Japanese version.

### Associations of intake of main staple foods with the health indices

There were associations between rice consumption and improvements in quality of life (vitality) and sleep quality (correlation coefficients: 0.129 and -0.133, respectively). Bread and noodle consumption were not associated with any of the health indices surveyed (Table 3).

**Table 3:** Correlations between consumption of rice or bread and the health indices.

Health index	Correlation coefficient		
	Rice	Bread	Noodle
SF-8 GH	0.037	0.048	0.031
SF-8 PF	-0.056	0.002	-0.008
SF-8 RP	0.018	0.012	-0.026
SF-8 BP	-0.071	0.047	-0.051
SF-8 VT	0.129*	0.006	0.004
SF-8 SF	0.012	-0.018	-0.077
SF-8 MH	0.053	0.071	-0.001
SF-8 RE	-0.040	0.070	-0.033
SF-8 PCS	-0.039	0.007	-0.021
SF-8 MCS	0.061	0.049	-0.020
BIS-11 Impulsive	-0.019	-0.098	-0.002
BIS-11 Deliberate	0.007	-0.055	0.022
BIS-11 Selfcont	-0.038	-0.049	0.054
BIS-11 Thinking	-0.089	-0.036	0.073
BIS-11 Sum	-0.045	-0.082	0.037
PHQ9	-0.026	-0.072	-0.030
PSQI-J	-0.133*	-0.034	0.036

SF-8 GH, general health; SF-8 PF, physical functioning; SF-8 RP, role—physical; SF-8 BP, bodily pain; SF-8 VT, vitality; SF-8 SF, social functioning; SF-8 MH, mental health; SF-8 RE, role—emotional; SF-8 PCS, physical component summary; SF-8 MCS, mental component summary: BIS-11, Barratt Impulsiveness Scale-11; BIS-11 Impulsive, subscale of impulsivity; BIS-11 Deliberate, subscale of lack of planning; BIS-11 Selfcont, subscale of lack of self-control; BIS-11 Thinking, subscale of non-planning impulsivity; BIS-11 sum, sum of subscales of BIS-11; PHQ9, Patient Health Questionnaire-9; PSQI-J, Pittsburgh Sleep Quality Index, Japanese version.

*p < 0.05,

**p < 0.01, Pearson’s correlation test

### Foods associated with rice intake

Typically, meals associated with the Japanese diet consist of rice and additional side dishes. To identify foods consumed with rice, the direct associations between rice intake and intake of other foods were analyzed. A Pearson’s association test between BDHQ scores for rice intake and other food intake indicated a low positive correlation between rice consumption and miso consumption (correlation coefficient: 0.275, Table 4). Low-fat fish, fermented soybeans, and green tea had correlations with rice intake (correlation coefficients: 0.118, 0.125, and 0.125, respectively; Table 4). Bread and noodle intake had low negative correlations with rice intake (correlation coefficients: -0.229 and -0.238, Table 4). Fresh vegetable and tomato intake had negative correlations with rice intake (correlation coefficients: -0.139 and -0.134, Table 4).

**Table 4:** Foods whose intake is correlated with rice intake.

Food item in BDHQ	Correlation efficient	Food item in BDHQ	Correlation efficient
Low-fat milk	-0.003	Radish or turnip	0.002
Milk	-0.101	Other root vegetables	-0.085
Chicken	-0.046	Tomato	-0.134*
Pork or beef	-0.065	Mushrooms	-0.079
Ham	0.095	Seaweeds	0.018
Liver	-0.023	Western confectionery	-0.072
Squid, octopus, shrimp, or shellfish	-0.044	Japanese confectionery	-0.016
Fish (with edible bones)	0.077	Rice crackers	0.012
Tuna (canned)	0.065	Ice cream	-0.087
Dried fish	0.083	Citrus	-0.032
High-fat fish	0.059	Persimmon or strawberry	-0.016
Low-fat fish	0.118*	Other fruits	-0.015
Egg	-0.001	Mayonnaise	-0.062
Tofu	-0.031	Bread	-0.229**
Fermented soybean	0.125*	Noodles	-0.238**
Potato	0.066	Green tea	0.125*
Pickled vegetables (greens, carrots, etc.)	0.020	Black tea or oolong tea	0.033
Pickled vegetables (light-colored vegetables)	0.071	Coffee	-0.105
Fresh vegetables	-0.139*	Coke	-0.100
Greens	-0.033	Fruit juice	-0.081
Cabbage	-0.091	Sugar	-0.004
Carrots or pumpkins	-0.022	Miso	0.275**

BDHQ, Brief-type Self-administered Diet History Questionnaire

* p < 0.05;

**p < 0.01, Pearson’s correlation test

### Indirect effects of the associations between rice intake and the health indices

**Fig 1:**
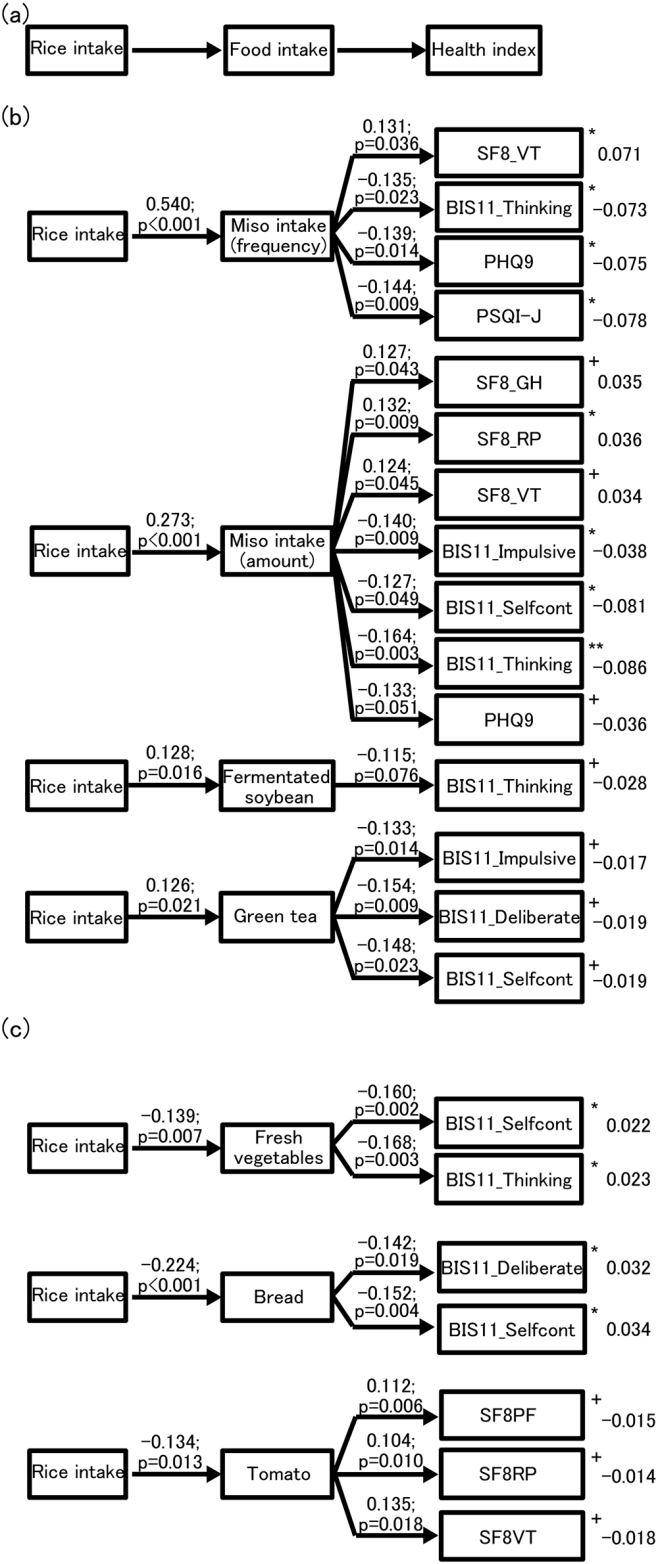
Indirect effects of the associations between rice intake and health. Structural equation modeling was performed to assess model fit considering “rice intake”, “foods”, and “health indices”. SF-8 GH, general health; SF-8 RP, role—physical; SF-8 VT, vitality; BIS-11, Barratt Impulsiveness Scale-11; BIS-11 Impulsive, subscale of impulsivity; BIS-11 Deliberate, subscale of lack of planning; BIS-11 Selfcont, subscale of lack of self-control; BIS-11 Thinking, subscale of non-planning impulsivity; PHQ9, Patient Health Questionnaire-9; PSQI-J, Pittsburgh Sleep Quality Index, Japanese version. (a) The structure of the model. (b) The fit of the model (rice intake, health indices, and foods whose intake was positively correlated with rice intake). The values on each arrow indicate path coefficients and p-values. Square sums of multiple correlation coefficients are shown to the right of the health indices. (c) The fit of the model (rice intake, health indices, and foods whose intake was negatively correlated with rice intake). The values on each arrow indicate path coefficients and p-values. Square sums of multiple correlation coefficients are shown to the right of the health indices. +p < 0.1; *p < 0.05; **p < 0.01.

To determine whether foods associated with rice intake indirectly contribute to the associations between rice intake and the health indices, path analysis using structural equation modeling was performed to evaluate the relationships between rice intake and the health indices (Fig 1a). Although we only found two direct correlations with rice intake, namely, quality of life (vitality) and sleep quality, additional associations were found between rice intake and health indices as mediators. Miso intake was identified as an indirect contributor to the association between rice intake and improvements in quality of life (vitality), impulsiveness (lack of forethought), depression, and sleep quality. The frequency of miso intake was found to be an indirect contributor to the association between rice intake and improvements in quality of life (role—physical) and impulsiveness (impulsive action, lack of self-control, and forethought). However, the effects of the association between rice intake and quality of life (vitality) as well as sleep quality were weaker than the direct effect. Thus, the direct effect of rice is greater than its indirect effect in these health indices. These causal relationships fit well in our model, and had total effects with statistical significance levels of 0.05. Frequency of miso intake affected the relationships between rice intake and improvements in quality of life (general health and vitality) and depression. Fermented soybean intake affected the relationship between rice intake and the improvement in impulsiveness, and green tea affected the relationship between rice intake and the improvement in impulsiveness (impulsive action, lack of planning, and lack of self-control). These causal relationships had a likelihood of fitting within our model and had statistical significance levels of 0.1 (Fig 1b). Analyses of the indirect effects of food intake indicated the presence of negative correlations with intake of rice, fresh vegetables, bread, and noodles. Fresh vegetables and bread had negative correlations with impulsiveness. There was a likelihood that tomato intake affected the correlation between rice intake and quality of life (physical functioning, physical role, and vitality) when statistical significance level of 0.1 was used (Fig 1c). There was no similar model fit for the consumption of noodles. The square sums of the multiple correlation coefficients were less than 0.1 for the all paths (right sides of Fig 1b and 1c). There was no model fit for low-fat fish, although low-fat fish intake correlated with rice intake as shown in the Table 4.

## Discussion

The direct and indirect analyses performed in this study indicated the presence of weak correlations. This may be due to the fact that individual foods have small contributions, as many food items were considered in this study. Nonetheless, the associations with statistical significance in the complex patterns of diet may be meaningful.

In Japanese cuisine, rice is a primary staple food and comprises a significant portion of the calories in the daily diet. We found direct correlations between rice intake and the health indices. Our results may support those of a previous study, wherein Yoneyama et al. reported that rice intake is associated with sleep quality [5]. Analysis of the indirect effects found in our study indicates that there may be a positive correlation between rice intake and health, which is modified by the consumption of other foods. Rice and miso consumption together might have a positive effect on health. Both rice and miso are included as top components in the Japanese Food Spinning Top [13], which is a guide for a healthy diet for the Japanese. There were correlations between other foods and health. However, these associations were weaker than those with rice-miso combinations. The absence of strong associations may be due to the fact that many of the additional foods assessed consisted of multiple ingredients, so that each food item did not have a large impact. Nevertheless, the relative correlations of the other foods investigated using the BDHQ suggest that these foods may be primary drivers of improvements in health or health maintenance.

A previous study by Yamamoto et al. evaluated the effects of Japanese model diets from 1960, 1975, 1990, and 2005 on health [14]. They prepared a feeding model based on the nutritional composition according to the diet from each year selected and administered the meals to mice. They concluded that the dietary pattern in 1975 may be the best for health because the mice fed the 1975 model diet had the longest lifespan and showed better capacities in learning and memory than the mice fed the other model diets. Moreover, a recent longitudinal study indicated that the 1975 Japanese diet may prevent cognitive decline [15]. Our study indicated that the combination of rice and miso may contribute to better physical and mental health, and, interestingly, rice and miso more frequently appear in the 1975 diet than in the 2005 diet. We conclude that our results support previous results that rice and miso are major components of the traditional Japanese diet that directly and indirectly positively affect health. Further research is needed to determine the specific roles these foods have on health. Our goal was to show the positive effects of the traditional Japanese cuisine, or Wa*shoku*. Therefore, the effects of the dietary patterns on health were analyzed, starting with rice intake.

Miso contains flavonoids, which are suggested to have benefits for physical health. For instance, flavonoids are thought to prevent cardiac disease, cancer, and osteoporosis [16], and to promote brain function, cognitive function, and learning and memory [17]. Green tea is rich in catechins, which are anti-oxidants and may help with glucose control [18], maintenance of cardiovascular function [19], and improvements in fat levels and blood pressure [20]. Although we have identified epidemiological associations between the consumption of specific foods and health, further physiological investigations may be necessary to understand the molecular mechanisms underlying the interactions of these foods with the body.

The modern diet is considered to be one of the main factors leading to the decline in population health in Japan [21]. However, the proportion of Japanese individuals with adult obesity is still low compared to that in other countries (Organization for Economic Cooperation and Development, 2015). Moreover, our study shows that bread and fresh vegetables and tomato, the consumption of which have increased after World War II [22, 23], are associated with improvements in impulsiveness, although they are negatively associated with rice consumption. This indicates that a rice-centered diet may have negative effects on impulsiveness. Although the present study investigated the merits of a rice-centered diet, exploring dietary patterns without bias from the traditional Japanese diet may be important in identifying a modern healthy Japanese diet. This is because Japanese dietary patterns have undergone large changes since World War II. The participants in the present study were 40 years of age or older and had consumed the traditional diet since 1975. Therefore, the foods identified as associated with improvements in health might correspond to representative foods in the participants’ diets throughout their lifespan. To elucidate the association between modern Japanese diets and improvements in health, the same investigation may be useful to assess younger individuals, around 20 years of age, who did not grow up with the traditional balanced diet of the 1975 era.

We found that sex is correlated with the health indices. This might be due to differences in energy metabolism or lifestyle. Subgroup comparisons in larger sample sizes may be informative.

In conclusion, the present study suggests an association between rice intake and physical and mental health, with indirect contributions from intakes of other foods as mediators. The foods that were associated with improvements in health were components of the traditional Japanese diet. Overall, rice, miso, fermented soybean, and green tea have been suggested as mediator of health in the traditional Japanese diet, or Washoku. We conclude that the contribution of the rice-centered Japanese diet to health may be explained by the effects of mediators, which are foods that have been familiar to the Japanese for a long time. Further research is required to assess the associations between modern foods and health and the biological mechanisms underlying these associations.
